# Endopolyploidy Changes with Age-Related Polyethism in the Honey Bee, *Apis mellifera*

**DOI:** 10.1371/journal.pone.0122208

**Published:** 2015-04-16

**Authors:** Juliana Rangel, Kim Strauss, Kaileah Seedorf, Carl E. Hjelmen, J. Spencer Johnston

**Affiliations:** Department of Entomology, Texas A&M University, College Station, Texas 77843, United States of America; Arizona State University, UNITED STATES

## Abstract

Honey bees (*Apis mellifera*) exhibit age polyethism, whereby female workers assume increasingly complex colony tasks as they age. While changes in DNA methylation accompany age polyethism, other DNA modifications accompanying age polyethism are less known. Changes in endopolyploidy (DNA amplification in the absence of cell division) with increased larval age are typical in many insect cells and are essential in adults for creating larger cells, more copies of essential loci, or greater storage capacity in secretory cells. However, changes in endopolyploidy with increased adult worker age and polyethism are unstudied. In this study, we examined endopolyploidy in honey bee workers ranging in age from newly emerged up to 55 days old. We found a nonsignificant increase in ploidy levels with age (*P* < 0.1) in the most highly endopolyploid secretory cells, the Malpighian tubules. All other cell types decreased ploidy levels with age. Endopolyploidy decreased the least amount (nonsignificant) in neural (brain) cells and the stinger (*P* < 0.1). There was a significant reduction of endopolyploidy with age in leg (*P* < 0.05) and thoracic (*P* < 0.001) muscles. Ploidy in thoracic muscle dropped from an average of 0.5 rounds of replication in newly emerged workers to essentially no rounds of replication (0.125) in the oldest workers. Ploidy reduction in flight muscle cells is likely due to the production of G1 (2C) nuclei by amitotic division in the multinucleate striated flight muscles that are essential to foragers, the oldest workers. We suggest that ploidy is constrained by the shape, size and makeup of the multinucleate striated muscle cells. Furthermore, the presence of multiple 2C nuclei might be optimal for cell function, while higher ploidy levels might be a dead-end strategy of some aging adult tissues, likely used to increase cell size and storage capacity in secretory cells.

## Introduction

Endopolyploidy is the cellular process of nuclear DNA amplification in the absence of typical mitotic cell division during the endocycle [1,2]. Endoreduplication is commonly observed in specialized plant and animal tissues, including a number of tissues in arthropods [3–9]. Age-related changes in endopolyploidy are often of interest because of their association with tumorogenesis [10]. However, the extent to which endopolyploidy levels change as a natural cell process related to aging is largely unstudied [11].

Endoreduplication plays an essential role during cell development and maintenance. Mutations that increase or decrease endoreduplication are often lethal to the organism [12,13]. The specific role of endoreduplication is not well established, however. Wu et al. [14] proposed that cells in specific tissues could employ endoreduplication to regulate transcription. In specialized mammalian tissues, such as those of the heart and liver, endoreduplication of somatic cells can be used to preserve energy under stressful conditions, or to upregulate specific organ functions [15]. Increasing ploidy levels in heart tissue leads to increased contractile protein expression, causing a switch from metabolically costly proteins to energy saving proteins. Furthermore, endopolyploidy appears to be utilized as a way to store nutrients in leaves and roots of plants, as well as in intestinal cells in *Drosophila* [2].

Bennett [16,17] argued that increased DNA content via endoreduplication causes “nucleotypic effects,” as genome size changes produced by endopolyploidy influence cell size and division rate [18,4,2,19], as well as gene expression and metabolic activity [18,4,20–22]. Endopolyploidy is also a contributor to sexual size dimorphism, as shown in members of the insect family Myrmecolacidae (Insecta: Strepsiptera) [6]. An example of the relationship of endopolyploidy and body size is observed in the nematode *Caenorhabditis elegans* [23]. In this species, because the fully developed worm has a fixed number of cells, its body size is regulated through endopolyploidy.

Endoreduplication occurs at different developmental stages in insects [18]. In *Drosophila melanogaster* the mechanisms by which the endocycle is regulated has been of particular interest [24,25], as many fully differentiated larval tissues, including those in the gut, epidermis, and trachea, exhibit endopolyploidy [1]. The endocycle is also active in adult tissues such as those present in the ovaries, Malpighian tubules, and salivary glands, all of which exhibit high levels of endoreduplication [8]. Tissue-specific differences in ploidy levels are also documented in the Hymenoptera [9,26–29], in which haploid males of all but the most basal lineages do not stay haploid, but rather undergo one round of endoreduplication to become functionally diploid [7]. Functionally diploid cells in mandibular, thoracic, and leg muscles of the haploid male bumble bee (*Bombus terrestris* L.) are comparable in size and function to those of the diploid female [7].

Scholes et al. [29] found that ploidy levels between and among worker castes of four highly polymorphic ant species are positively related to worker size, suggesting that worker task performance might gain an adaptive benefit from endoreduplication of certain tissues. For example, organs associated with digestive and exocrine functions in the ant *Dinoponera australis* were recently shown to have higher levels of endopolyploidy than organs in the reproductive, muscular, and neural system, suggesting that increased cell size via endoreduplication in functional organs might occur during caste-based task allocations [9]. The suggestion that endopolyploidy plays a role in caste-based task allocation raises an interesting question: is it possible that endopolyploidy changes occur in association with caste-based reproductive division of labor in the worker caste of the honey bee, *Apis mellifera*?

In honey bees, newly emerged female workers exhibit age polyethism, the allocation of tasks of increasing complexity to workers as they age. For example, newly emerged workers stay in the nest as “nurses” that tend the queen and feed the brood, while older workers assume increasingly risky roles, eventually flying out of the nest as they become foragers and scouts [30]. Interestingly in honey bees, methylation of DNA decreases with worker age and behavioral subcastes [31]. Whether this drop in DNA methylation is coincident with changes in endopolyploidy as workers age is entirely unknown.

In this study, we hypothesized that endopolyploidy would change in honey bees to meet the roles and demands of different tissue types as workers transition from one age-related task to the next. To test this hypothesis, we compared tissue-specific ploidy levels over a wide range of worker ages in the stinger, as well as in three tissues types: muscle (thorax and leg), neural (brain) and digestive/endocrine (Malpighian tubules). Our results show that endopolyploidy levels change with worker age in a tissue-specific manner. We discuss the implication of our findings with regard to the role of endopolyploidy in eusocial insect development.

## Methods

### Honey bee collection

Honey bee workers were collected from two source colonies housed at the John G. and Janice Thomas Honey Bee Facility at Texas A&M University in College Station, Texas (30° 38' 31.9" N, 96° 27' 39.4" W). Each source colony contained approximately fifteen thousand workers and one naturally mated Italian queen. To determine the exact day on which workers emerged from their cells, we collected from each colony a frame of sealed pupae approaching emergence [30]. We then placed each frame of sealed brood in a “nucleus” box along with a frame of food (honey and pollen) in an incubator kept at 34°C, the average temperature around a colony’s brood area [30,32]. Both boxes were checked daily until adult workers started to emerge from cells. We collected approximately 200 0-day-old workers from each nucleus box and placed a color mark on their thorax, using a different color for each colony as described previously by Seeley [33]. A subsample of emerging workers was sacrificed by freezing at -80°C (see below). The remaining painted workers were returned to their original source colonies and allowed to mature naturally. We collected labeled workers on eight subsequent days ranging from 1 to 55 days post-emergence and sacrificed them by freezing at -80°C.

Frozen workers were subsequently thawed and prepared for dissection following standard procedures [34]. From each worker we dissected out the stinger, thoracic and leg muscle tissue, brain tissue, and Malpighian tubules tissue. We collected a total of two samples of each adult tissue and age, except for workers collected 27 and 34 days post emergence, for which we only collected one sample, and workers collected six days post emergence, for which we collected three samples. We were only able to collect one Malpighian tubules tissue on day 13 post emergence (Table 1).

**Table 1:** Ploidy levels for five tissues from honey bee (*Apis mellifera*) workers at different days post emergence (0 to 55 days of age).

**Tissue**	**Age (days)**	**0**	**1**	**3**	**6**	**13**	**20**	**27**	**34**	**55**
Brain	*N*	2	2	2	3	2	2	1	1	2
	Ploidy	0.3	0.25	0.18	0.21	0.26	0.21	0.17	0.18	0.19
	SE	0.02	0.04	0.01	0.02	0.03	0	n.a.	n.a.	0
Flight muscle	*N*	2	2	2	3	2	2	1	1	2
	Ploidy	0.59	0.56	0.56	0.53	0.35	0.4	0.23	0.19	0.13
	SE	0.06	0.03	0.13	0.14	0.17	0.08	n.a.	n.a.	0
Leg muscle	*N*	2	2	2	3	2	2	1	1	2
	Ploidy	0.89	0.97	0.97	0.79	0.78	0.75	0.61	0.79	0.73
	SE	0.05	0.07	0.09	0.03	0.02	0.22	n.a.	n.a.	0.08
Malpighian tubules	*N*	2	2	2	3	1	2	1	1	2
	Ploidy	2.16	1.9	2.13	2.01	2.17	2.21	2.19	2.6	2.26
	SE	0	0.12	0.17	0.13	n.a.	0.11	n.a.	n.a.	0.21
Stinger	*N*	2	2	2	3	2	2	1	1	2
	Ploidy	1.27	1.25	1.06	1.14	1.27	1.37	1.32	1.08	0.96
	SE	0.05	0.03	0.11	0.06	0.08	0.03	n.a.	n.a.	0.07

### Flow cytometric ploidy determination

**Fig 1:**
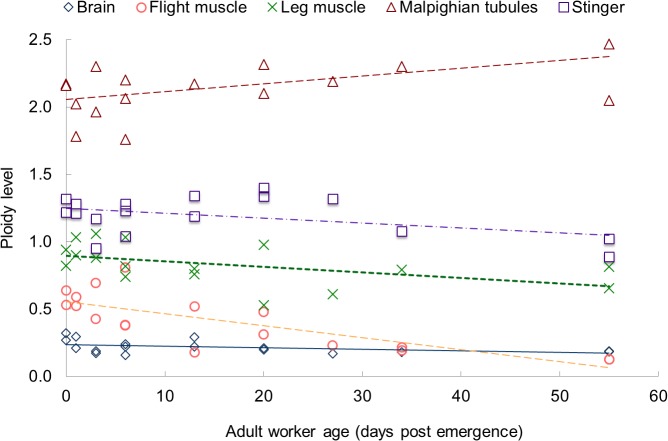
Least square estimates of mean ploidy levels post-emergence (intercept), and change of ploidy over time (slope) for five different worker honey bee (*Apis mellifera*) tissues: Brain, thoracic (flight) muscle, leg muscle, Malpighian tubules, and stinger. Adult honey bee workers were collected at different days post emergence (See *Methods*for details).

Cell nuclei were isolated from each worker’s honey bee stinger and tissue samples as described in Scholes et al. [9]. In brief, tissues were isolated and then ground in 1 mL of Galbraith buffer (45 mM magnesium chloride, 30 mM sodium citrate, 20 mM 4-morpho-G line propane sulfonate, and Triton X-100 (1 mg/mL)) [35] using ten gentle strokes in a 2 mL Kontes Dounce homogenizer using the “A” pestle. The tissues were then filtered using a 40U nylon mesh to remove debris. The resultant solution was brought to 1 mL with additional buffer and was stained using 25 μL Propidium Iodide for 20 minutes in the dark at 4°C. A Partec Cyflow cytometer (Partec America, Swedesboro, NJ) was used to score relative fluorescence and thereby the amount of DNA in the nuclei isolated from each stinger and from each of the other tissue samples. The cytometer was used to score number of nuclei based on amount of PI fluorescence. Histograms were then produced with multiple discrete peaks that represented different fluorescence levels associated with different ploidy levels (Fig 1). The cytometer was adjusted to show diploid (2C) nuclei at channel 25, which made it possible to display 2C, 4C, 8C, 16C, and 32C ploidy levels. The number of nuclei under each ploidy peak was scored using standard Partec software. The few nuclei with ploidy of 64C+ accumulated and were counted in channel 500.

### Ploidy Level

Rather than report the count as a weighted average of 2, 4, 8, 16, 32 and 64 ploid nuclei, which produces mean values that are difficult to interpret in terms of replication cycles, the average endoreduplication cycles per nucleus in each sample were calculated as in Barow and Meister [36] using the following equation:

$$Ploidy=\frac{\left((0\times 2C)+(1\times 4C)+(2\times 8C)+(3\times 16C)+(4\times 32C)+(5\times 64C)\right)}{\left(2C+4C+8C+16C+32C+64C\right)}$$

The ploidy level is 0.0 for unreplicated diploid cells at G1, 1.0 for fully replicated G2 cells, and increases incrementally with each successive round of endoreduplication.

### Statistical Analysis

Changes in ploidy levels for each honey bee worker tissue type were compared over time using a test for equal slopes with an analysis of covariance (ANCOVA) using the statistical software SAS 9.4 (SAS Institute Inc., Cary, NC). Our model contained an interaction term of age post emergence and tissue type, with day and hive number treated as random effects, and tissue as a fixed effect (Model: Y_ijk_ = μ + τ_i_ + β_j_ + τβ_ij_ + ε_ijk_, in which μ = mean, τ = day effect, β = tissue effect, τβ = interaction effect, and ε_ijk_ = error in model). A least square fit of the endopolyploid level as a function of worker age produced least square estimates of ploidy levels. Test of significance of differences in ploidy levels per tissue were conducted using the “pdiff” option in PROC GLM. Regression analyses were performed to produce least square estimates of the intercept (ploidy level at day 0) and slope (ploidy change per day) for each tissue. We set the level of statistical significance at α = 0.05, but report the p level when *P* < 0.10 and when *P* < 0.001.

## Results

Ploidy levels were determined for 84 samples from 9 age classes (0- to 55-day-old honey bee workers) for five tissue types: brain, thoracic muscle, leg muscle, Malpighian tubules, and stinger with 1 to 3 replicates depending on the number of workers recovered for each age class (Table 1). The level of endopolyploidy in newly emerged workers (intercept) was highly significantly greater than 0.0 (diploid) for all tissues (*P* < 0.001). A regression analysis produced a least square estimate of the ploidy change per day (slope). Ploidy levels changed with age for all tissue types, although the change was not always significant and was different for each tissue (Table 2).

**Table 2:** Least squares estimates of endopolyploid levels in newly emerged honey bee workers (i.e., "Intercept") and rate of change of endopolyploidy per day (i.e., "Slope") for the five tissues studied.

**Tissue**	**Intercept** ***	**Slope**	**R** ^2^	***P*-value**
Brain	0.237	-0.0012	0.45	0.07
Flight Muscle	0.556	-0.0088***	0.59	0.0003
Leg Muscle	0.897	-0.0041*	0.24	0.04
Malpighian tubules	2.056	0.0057	0.23	0.06
Stinger	1.25	-0.0036	0.19	0.08

* *P* < 0.05

*** *P* < 0.001

The initial levels of endoreduplication of 0-day-old workers, as well as the least squares regression change over time are shown graphically in Fig 1. Endopolyploidy increased only in the highly endopolyploid secretory tissue of the Malpighian tubules with 2.16 rounds of replication on average in newly emerged workers. The Malpighian tubules tissue showed an increase in ploidy with age, although that increase was not significant (+ 0.0057 / day; *P* < 0.1). Neural tissues (brain) had the lowest level of endoreduplication (0.3 rounds of replication on average in newly emerged workers) and a very slight decrease in endopolyploidy level with age, which was not significant (-0.0012/day; *P* < 0.1). All other tissues had intermediate levels of endoreduplication and decreased endopolyploidy with age, examples of which can be seen in S1 Fig. The second most endoreduplicated tissues were those of the stinger (1.3 rounds of replication on average in newly emerged workers), although the decrease in level of endoreduplication with age was not significant (-0.0036; *P* < 0.1).

**Fig 2:**
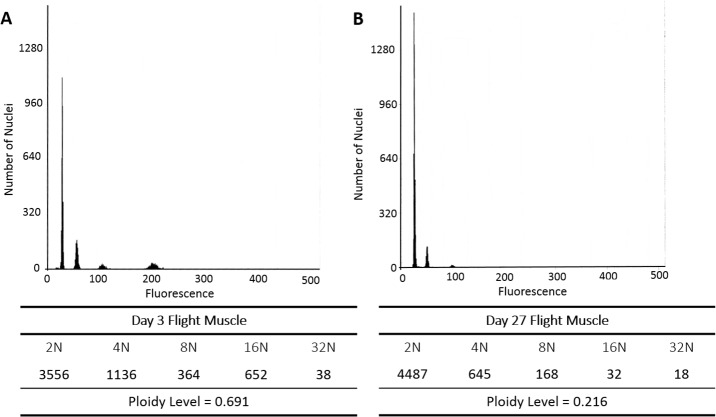
Histograms showing fluorescence peaks centered at mean fluorescence channels 25, 50, 100, 200, and 400 produced by 2N, 4N, 8N, 16N and 32N nuclei isolated from flight muscle of (A) a 3-day-old and (B) a 27-day-old worker bee. The count of nuclei under each peak and the average ploidy level are given immediately below each histogram. The reported significant reduction of ploidy with age (*P* < 0.01) is reflected in the reduced 8N peak and 16N peak in the older bee.

In contrast, ploidy levels in muscle tissues decreased significantly with age. The leg muscles were replicated through almost one round (0.90) at emergence, and that level decreased significantly as workers aged (-0.0041; *P* < 0.05). The muscles of the thorax (i.e., flight muscles) had the second smallest levels of endoreduplication (0.59) at emergence, yet showed the most significant reduction of endopolyploidy with age (-0.0088; *P* < 0.001). Fig 2 shows an example of ploidy reduction in flight muscle from 0.69 at age 3 to 0.22 at age 27 post emergence. By 55 days post emergence, almost all of the cell nuclei of thoracic muscles were unreplicated (diploid), with average endoreduplication levels below that of even the neural tissues (Fig 1).

## Discussion

We report the first estimates of endopolyploidy levels for tissues in newly emerged honey bee workers and provide the first evidence that endopolyploidy levels change significantly as workers age. Prior to our study, there was almost no information on naturally occurring change of ploidy levels over time [1,2,21]. In insects, it was not known if ploidy levels were established by the end of pupation, increased shortly after emergence of the adult, or changed in any direction with adult age.

Using the honey bee as a model to study endoreduplication in eusocial insects, we now know that ploidy levels can change over time and are tissue specific. In accordance with previous findings [29], Malpighian tubules exhibited relatively high ploidy levels, which increased with worker age, albeit not significantly (*P* < 0.1). This relatively high ploidy in the Malpighian tubules highlights the essential role of endopolyploidy in insect secretory cells [9]. The other cells types showed either a small nonsignificant decrease in endopolyploidy (i.e., tissues of the brain and stinger), or a significant decrease in endopolyploidy (i.e., tissues of the leg and thoracic muscle) as workers aged.

The observed decrease in ploidy levels of some tissues as workers aged, in particular the highly significant decrease in ploidy in thoracic muscle tissue, was unexpected. Even though this is the first study looking at endopolyploidy levels in the age-related castes in eusocial insects, previous studies have indicated that endoreduplication might be important in the upregulation of transcription [14], organ function [15], and nutrient storage [2]. In Lepidoptera ploidy levels in flight muscle nuclei increase with each stage of larval development, although a decrease in ploidy level in epidermal cells has also been reported for the last larval instar [37].

One proposed benefit to decreasing ploidy level with age is the warding off of tumors. Polyploid cells are a precursor to aneuploid cells involved in some cancers [10]. By disallowing the endoreduplication with age in certain tissue types, the organism could prevent the proliferation of tumor cells that become a risk with increased age. Ploidy changes over time have been reported for the spermatheca of honey bee queens by Peres et al. [38], who suggest that an increase in ploidy is a mechanism for coping with increased reactive oxygen species over the 1.5-year lifespan of the queens examined. The worker honey bee lifespan is much shorter (i.e., 30–60 days post emergence depending on the season [30]), yet a small increase in ploidy level (*P* < 0.1) was observed for the Malpighian tubules. One might therefore expect ploidy to increase at a relatively fast rate in muscle cells, particularly in the flight muscle of aging workers where cell size would be exposed to the highest number of reactive oxygen species as a consequence of the demands of flight [39–41]. What we find, however, is that the many reasons suggested for an increase in endopolyploidy, including protection against damage due to oxidative stress, increased cells size and increased transcript copies due to increased copies of DNA strands, simply do not apply in honey bee flight muscle cells.

Existing proposals regarding the benefits of endopolyploidy in muscle cells may not fit in the honey bee model due to the nature of their multinucleate muscle cells [42]. The lack of fit is not because the multinucleate structure affects the ploidy score. Instead, the nuclei scored here are single isolated nuclei, not multiple nuclei. Therefore, the significant decreases in endopolyploidy may be more likely associated with age-related amitosis that over time produces more nuclei, but with lower ploidy levels in the multinucleate striated muscle cells [43]. The observed reduction of ploidy in muscle cells may reflect constraint on genome size due to the elongated cells of striated muscle.

A large number of studies have shown that cell size increases with an increase in DNA amount [4,17,18]. It is less well documented, however, whether the opposite may also be true, and DNA amount can be constrained by limited cell size, as documented in dipteran development by Schmidt-Ott et al. [44]. Similar cell size constraint might be expected in insect striated flight muscle cells, in which up to 40% of the total volume can be composed of mitochondria [44]. Size-related constraints on genome size of nuclei could account for the nuclear morphology during amitotic nuclear divisions in striated flight muscle. These divisions are characterized by nuclear size increase only along the long axis with subsequent symmetric division in which the DNA ploidy is reduced and the amount of DNA is distributed equally into daughter nuclei [45–49].

In conclusion, this study provides the first evidence of changes in endopolyploidy levels in different tissues of honey bee workers as they age. Our findings suggest that ploidy levels may change over time in all tissues examined, with a nonsignificant increase in ploidy only observed in issues of the Malpighian tubules. A decrease in ploidy, albeit non significant, was observed in tissues of the brain and stinger. In contrast, we found a surprisingly significant decrease in ploidy in tissues of the leg and the thoracic muscles, which are those associated with flight. We propose that the decrease in ploidy in flight muscle tissues as workers age might be because the size of nuclei in these cells are constrained by the shape and the constitution of striated muscle. The nuclei undergo amitotic divisions and become increasingly multinucleate, rather than highly polyploid. Further studies should elucidate the mechanisms underlying endoreduplication in tissues of other eusocial insects, and test whether the same patterns emerge in species that exhibit age-polyethism versus those in which tasks are allocated to members of specific morphological castes.

## Supporting Information

S1 Fig Histograms showing successive fluorescence peaks produced by 2N, 4N, 8N, 16N and 32N nuclei and showing vertical lines and bars that establish the range of the corresponding statistical gates RN1, RN2, RN3, etc., that count and output the total number of nuclei in each peak. The ploidy level is calculated as a weighted average, as described in the text, and is based on the counts at each ploidy level times the number of rounds of replication (i.e., 0,1,2,3, etc.) that produced the amount DNA in the nuclei scored as 2N, 4N, and successive ploidy peaks. Panels A, B, and C are histograms based on different samples prepared from different tissues of worker bees of known age, as given with each successive histogram (below panels A through E). Panels B and E show ploidy change in flight muscle over a wider age range than shown in Fig 1 in the main article. Panel F is for Malpighian tissue of a 55-day-old worker and was selected to show the highest ploidy level we observed during sampling.
(TIFF)
