# Supplementary figures and images for: Endopolyploidy Changes with Age-Related Polyethism in the Honey Bee, Apis mellifera
Source: PLoS One. 2015 Apr 16;10(4):e0122208. doi: 10.1371/journal.pone.0122208 (PMC4400096; doi:10.1371/journal.pone.0122208)

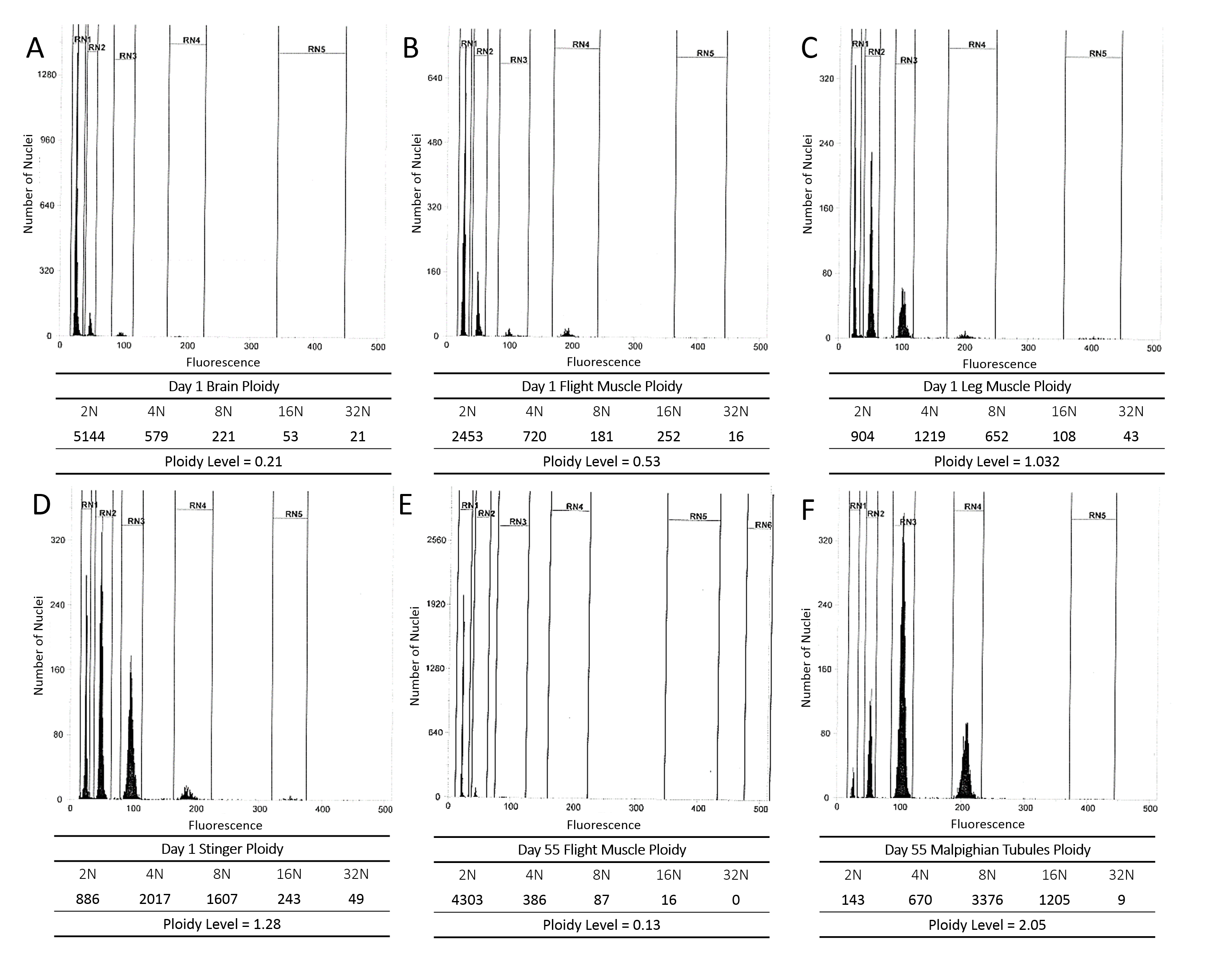

Supplement: S1 Fig — The ploidy level is calculated as a weighted average, as described in the text, and is based on the counts at each ploidy level times the number of rounds of replication (i.e., 0,1,2,3, etc.) that produced the amount DNA in the nuclei scored as 2N, 4N, and successive ploidy peaks. Panels A, B, and C are histograms based on different samples prepared from different tissues of worker bees of known age, as given with each successive histogram (below panels A through E). Panels B and E show ploidy change in flight muscle over a wider age range than shown in Fig 1 in the main article. Panel F is for Malpighian tissue of a 55-day-old worker and was selected to show the highest ploidy level we observed during sampling. (TIFF) [file pone.0122208.s001.tiff]
